# Supplementary material for: EFA6 regulates selective polarised transport and axon regeneration from the axon initial segment
Source: J Cell Sci. 2017 Nov 1;130(21):3663–75. doi: 10.1242/jcs.207423 (PMC5702059; doi:10.1242/jcs.207423)
Supplement: Supplementary information [file joces-130-207423-s1.pdf]

Supplementary Figures

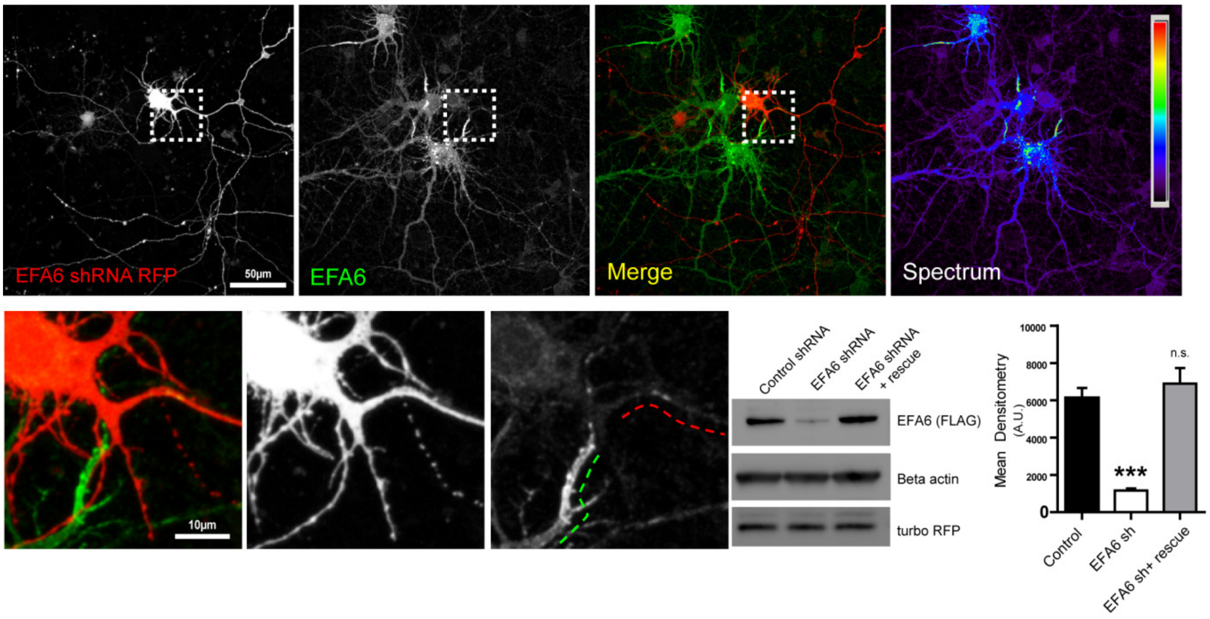

Fig. S1. Validation of shRNA targeting EFA6. E18 cortical neurons transfected with shRNA targeting EFA6 (red, RFP) at 10DIV, fixed at 14DIV and immunolabelled for EFA6. Lower panels are high magnification of the region indicated by the dashed box in the upper panels, showing low levels of EFA6 in the initial part of the axon of a transfected cell (red dashes), compared with high levels of EFA6 in the untransfected cell (dashed green lines). (**B**) Validation of EFA6 shRNA by western blotting. Blots show lysate from PC12 cells transfected with either rat EFA6-FLAG and or rat EFA6- FLAG plus human EFA6-FLAG (as an shRNA resistant rescue plasmid) together with control or EFA6 shRNA. Graph is quantification of silencing effect by densitometry n=3. ***p=0.0002, f=25.8, ANOVA and Bonferroni's test. Error bars are SEM.

Journal of Cell Science • Supplementary information

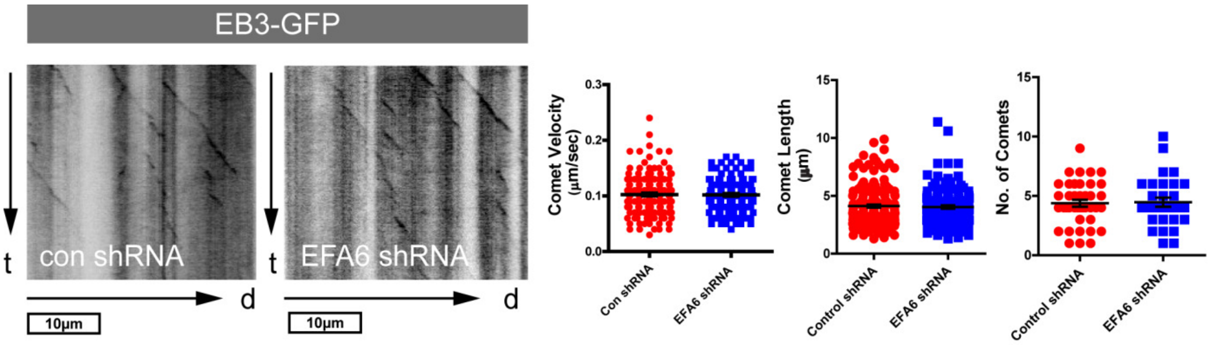

Fig. S2. EFA6 silencing does not alter axon microtubule dynamics. Representative kymographs and analysis of EB3-GFP comets in axons of E18 14DIV cortical neurons co-expressing EB3-GFP and control or EFA6 shRNA. n = 30 and 40 neurons. EB3-GFP localised strongly to the AIS, however comets were undetectable here in cells expressing either control or EFA6 shRNA (see movies S1 and S2). Error bars are SEM.

Journal of Cell Science • Supplementary information

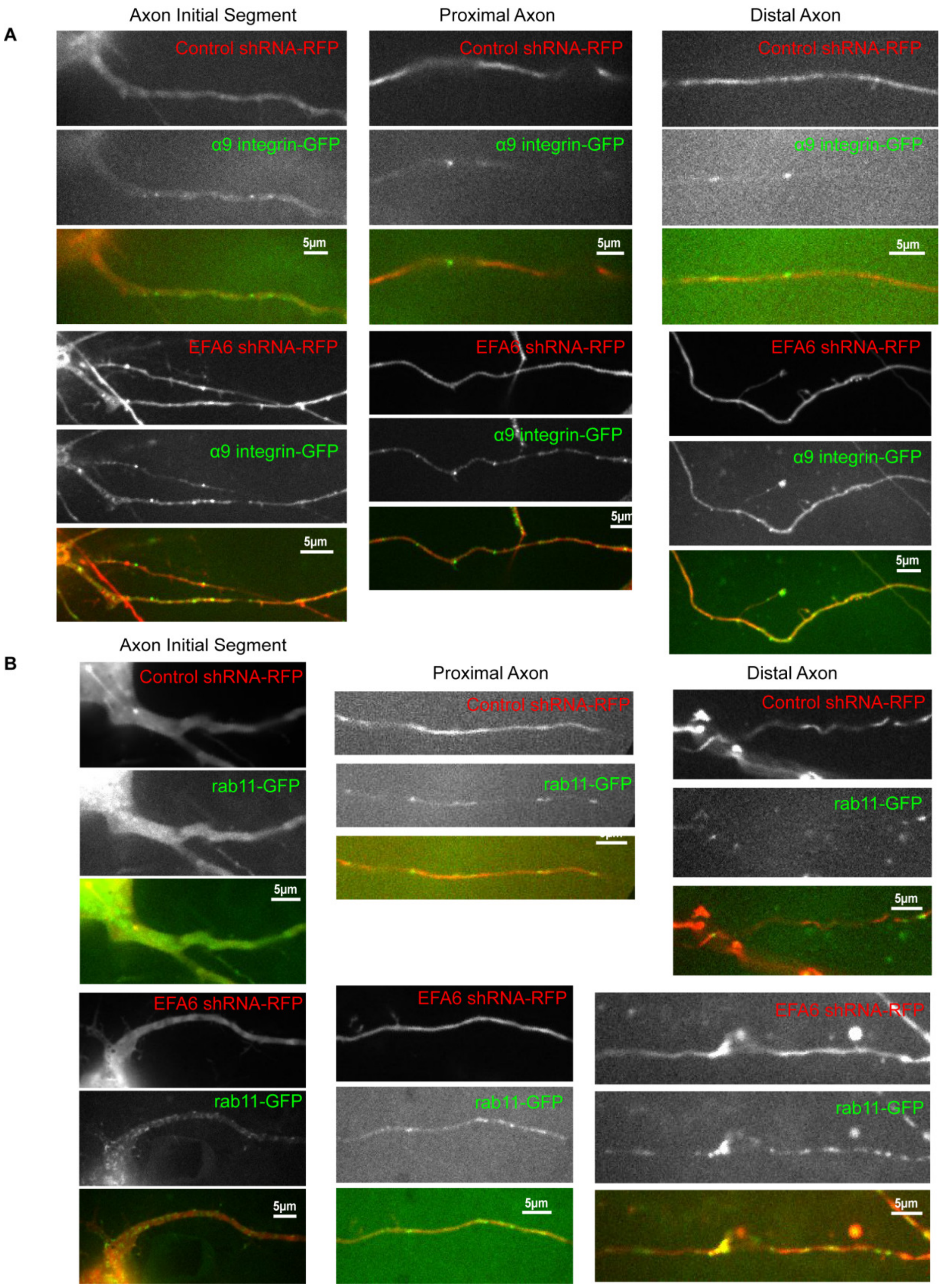

Fig. S3. Single frame images of movies used to analyse axon endosome dynamics. Images show sections of the initial, proximal or distal part of axons, expressing α9 integrin-GFP or rab11-GFP together with either control or EFA6 shRNA. (**A**) Axons of E18 14-17DIV cortical neurons transfected at 10DIV with control or EFA6-shRNA, and co-transfected with α9 integrin-GFP. (**B**) Axons of E18 14-17DIV cortical neurons transfected at 10DIV with control or EFA6-shRNA, and co-transfected with rab11-GFP.

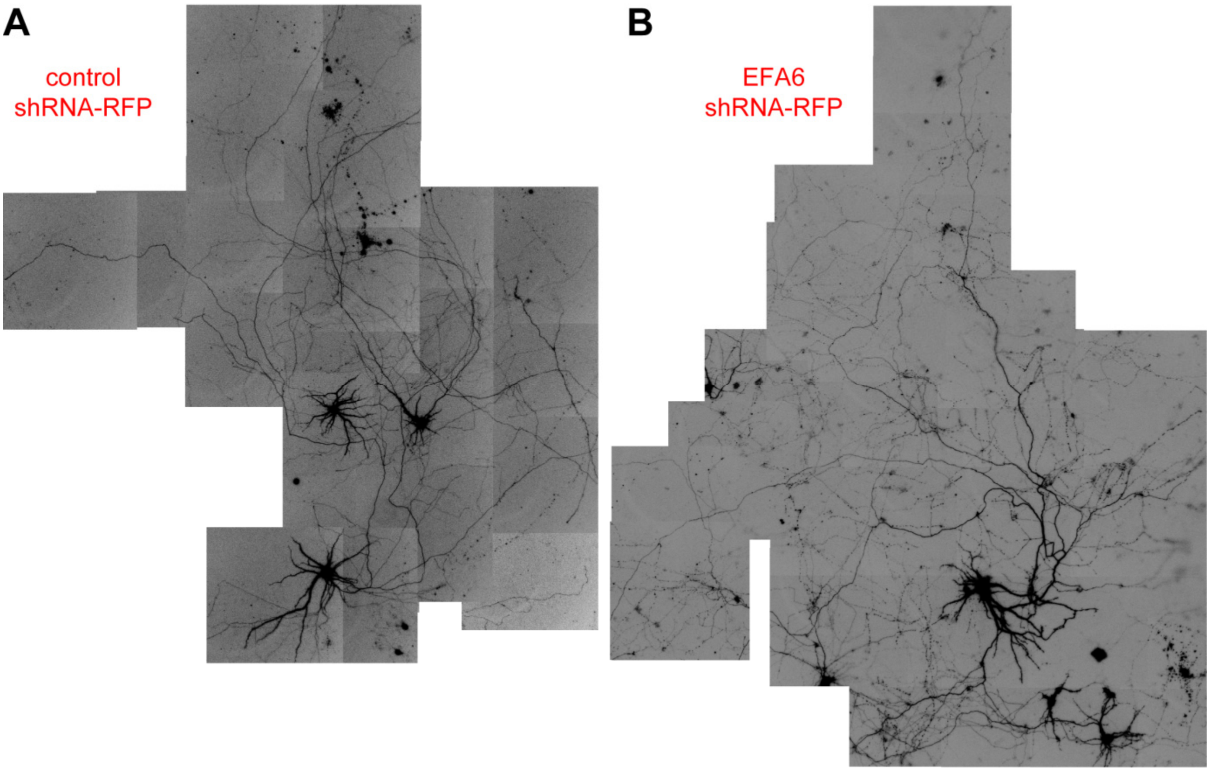

Fig. S4. Examples of control- or EFA6-shRNA expressing neurons used for axotomy experiments. (**A**) Example of cortical neurons transfected with control shRNA-RFP (signal is RFP) at E18, 10DIV, used for axotomy experiments at E18, 14-17DIV. (**B**) Example of cortical neurons transfected with EFA6 shRNA-RFP (signal is RFP) at E18, 10DIV, used for axotomy experiments at E18, 14-17DIV.

Journal of Cell Science • Supplementary information

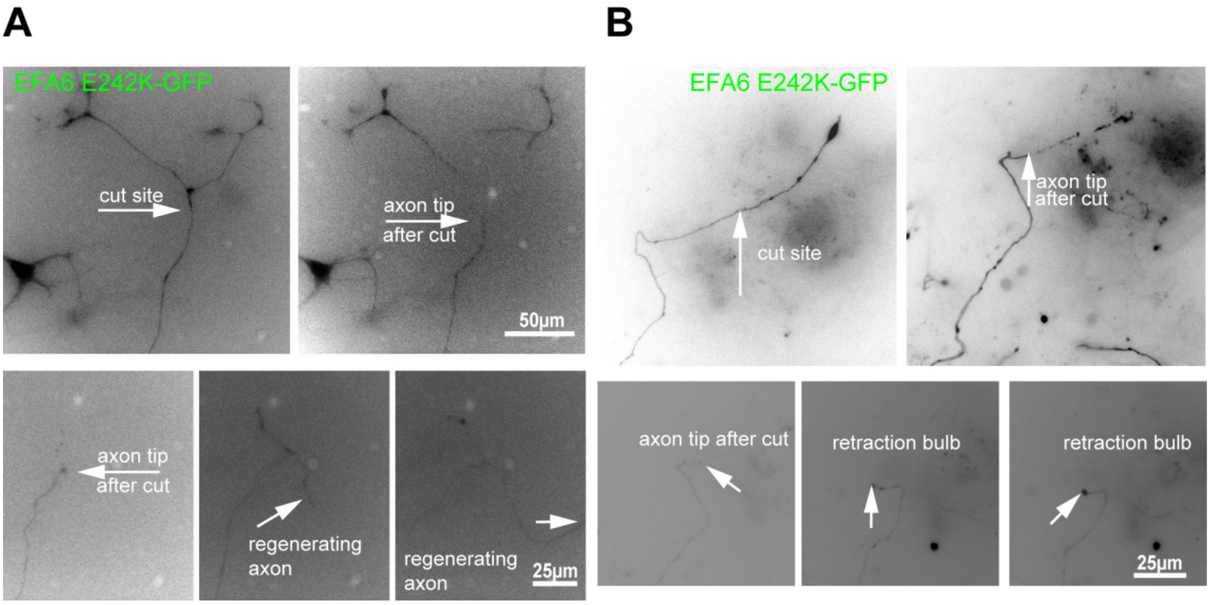

Fig. S5. DRG neurons expressing EFA6 with inactive GEF domain (EFA6 E242K). (**A**) Example of successful regeneration after axotomy, DRG neuron expressing EFA6 E242K. (**B**) Example of regeneration failure after axotomy, DRG neuron expressing EFA6 E242K. Expression of EFA6 E242K allows 50.5% of axons to regenerate their growth cones after axotomy.

Journal of Cell Science • Supplementary information

Movies

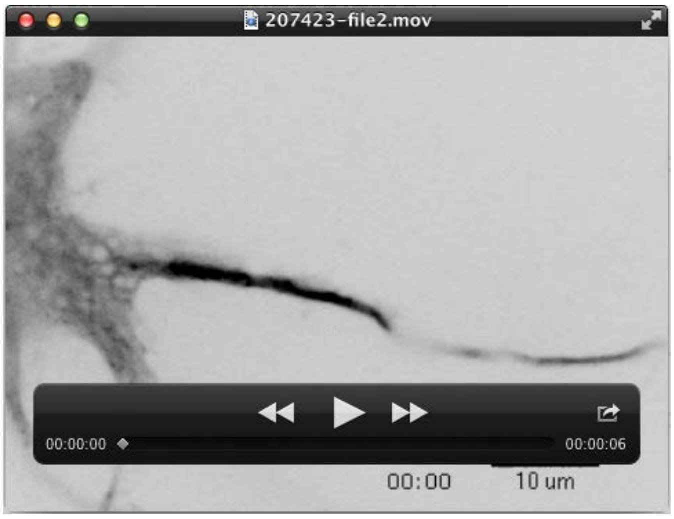

Movie 1. EB3-GFP localised to the axon initial segment and cell body (comets) of E18 15DIV cortical neuron, also expressing control shRNA.

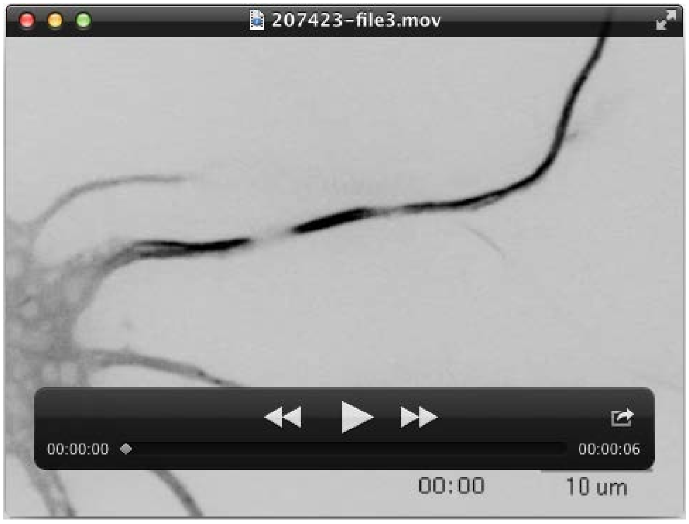

Movie 2. EB3-GFP localised to the axon initial segment and cell body (comets) of E18 15DIV cortical neuron, also expressing shRNA targeting EFA6. The apparent break in the axon is a section of axon out of the plane of focus.

Journal of Cell Science • Supplementary information

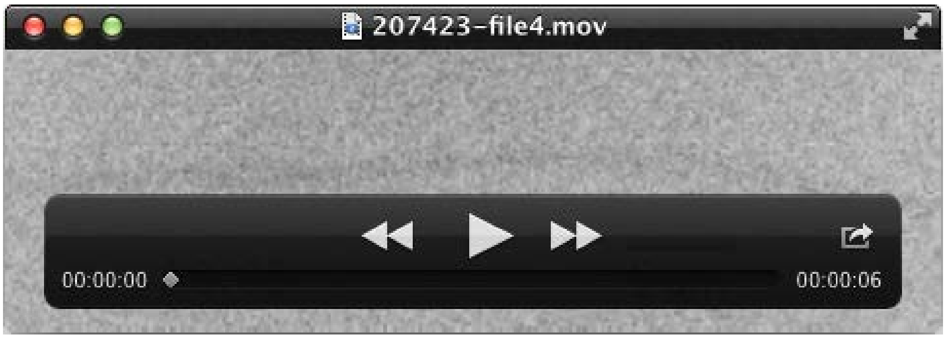

Movie 3. α9 integrin axon transport in the distal section of an axon also expressing control shRNA (see fig. S2). Movement to the left hand side is retrograde, right hand side anterograde.

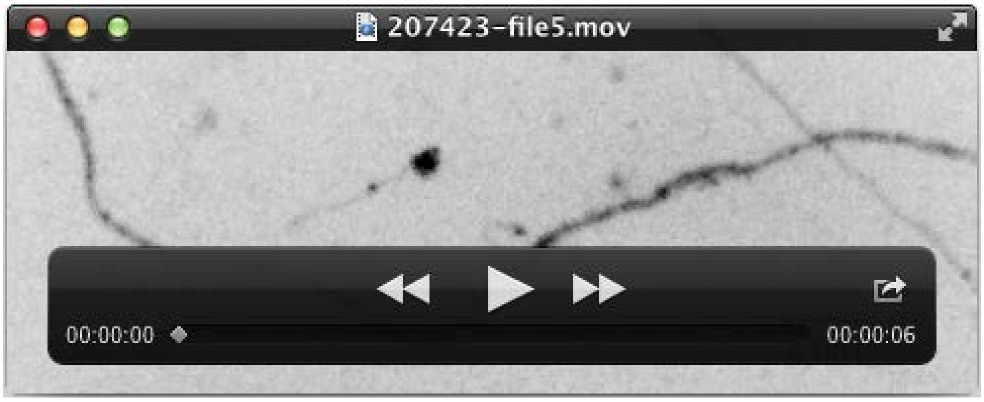

Movie 4. α9 integrin axon transport in the distal section of an axon also expressing shRNA targeting EFA6 (see fig. S2). Movement to the left hand side is retrograde, right hand side anterograde.

Journal of Cell Science • Supplementary information

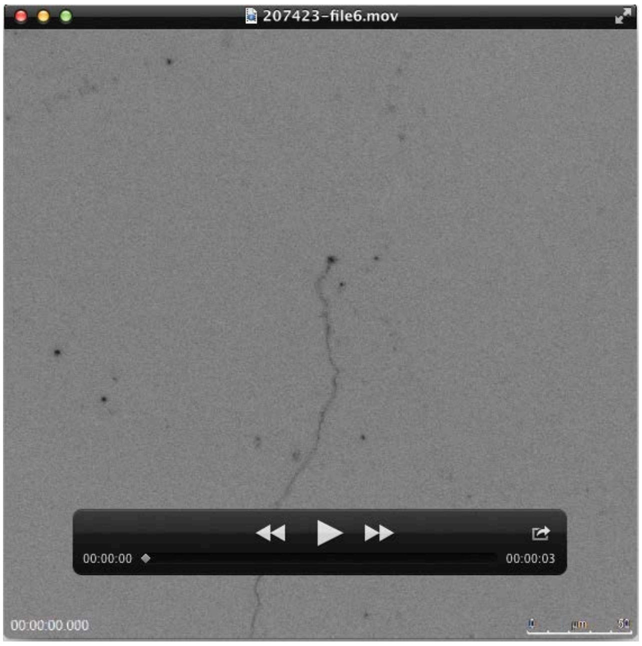

Movie 5. Axon regeneration after laser axotomy of E18 15DIV cortical neuron expressing control shRNA.

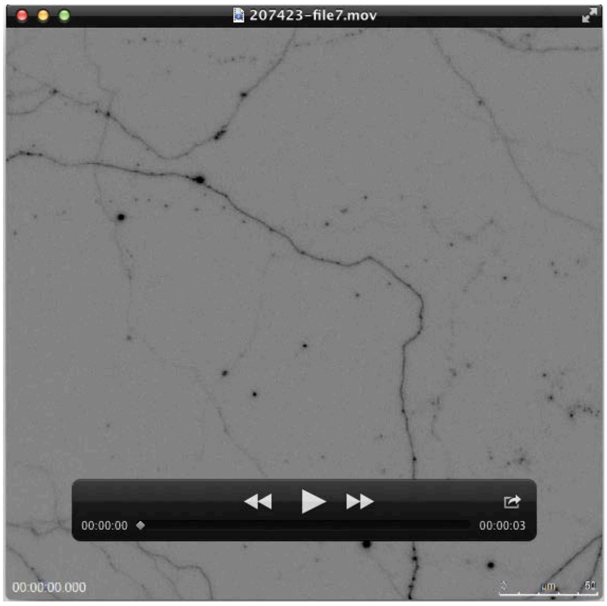

Movie 6. Axon regeneration after laser axotomy of E18 15DIV cortical neuron expressing shRNA targeting EFA6.

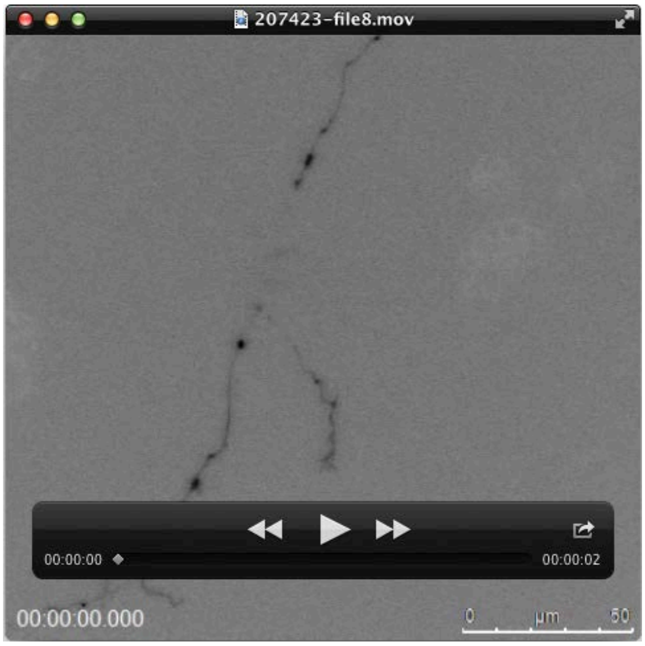

Movie 7. Axon regeneration after laser axotomy of adult DRG neuron expressing GFP.

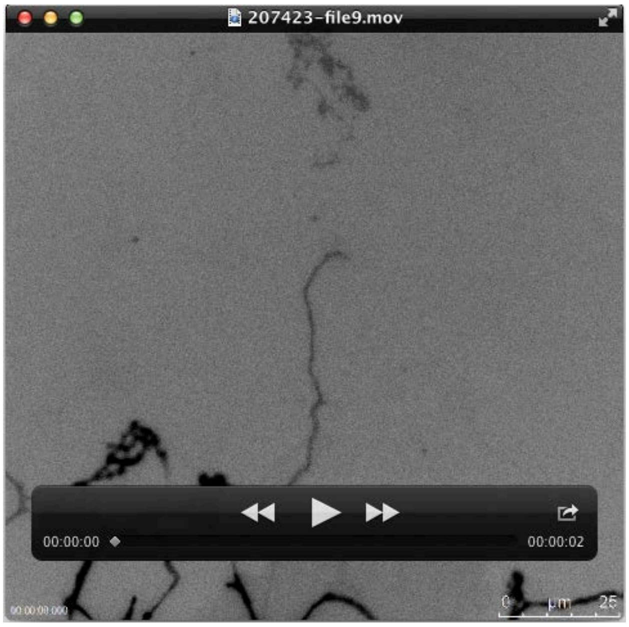

Movie 8. Failed axon regeneration after laser axotomy of adult DRG neuron overexpressing EFA6-GFP.

Journal of Cell Science • Supplementary information
